# Personality Is Reflected in the Brain's Intrinsic Functional Architecture

**DOI:** 10.1371/journal.pone.0027633

**Published:** 2011-11-30

**Authors:** Jonathan S. Adelstein, Zarrar Shehzad, Maarten Mennes, Colin G. DeYoung, Xi-Nian Zuo, Clare Kelly, Daniel S. Margulies, Aaron Bloomfield, Jeremy R. Gray, F. Xavier Castellanos, Michael P. Milham

**Affiliations:** 1 Phyllis Green and Randolph Cōwen Institute for Pediatric Neuroscience at the NYU Child Study Center, New York University Langone Medical Center, New York, New York, United States of America; 2 Department of Psychology, Yale University, New Haven, Connecticut, United States of America; 3 Department of Psychology, University of Minnesota, Minneapolis, Minnesota, United States of America; 4 Laboratory for Functional Connectome and Development, Key Laboratory of Behavioral Sciences, Institute of Psychology, Chinese Academy of Sciences, Beijing, China; 5 Max Planck Institute for Human Cognitive and Brain Sciences, Leipzig, Germany; 6 Mind and Brain Institute, Humboldt University, Berlin, Germany; 7 Nathan Kline Institute for Psychiatric Research, Orangeburg, New York, United States of America; 8 Center for the Developing Brain, Child Mind Institute, New York, New York, United States of America; Cuban Neuroscience Center, Cuba

## Abstract

Personality describes persistent human behavioral responses to broad classes of environmental stimuli. Investigating how personality traits are reflected in the brain's functional architecture is challenging, in part due to the difficulty of designing appropriate task probes. Resting-state functional connectivity (RSFC) can detect intrinsic activation patterns without relying on any specific task. Here we use RSFC to investigate the neural correlates of the five-factor personality domains. Based on seed regions placed within two cognitive and affective ‘hubs’ in the brain—the anterior cingulate and precuneus—each domain of personality predicted RSFC with a unique pattern of brain regions. These patterns corresponded with functional subdivisions responsible for cognitive and affective processing such as motivation, empathy and future-oriented thinking. Neuroticism and Extraversion, the two most widely studied of the five constructs, predicted connectivity between seed regions and the dorsomedial prefrontal cortex and lateral paralimbic regions, respectively. These areas are associated with emotional regulation, self-evaluation and reward, consistent with the trait qualities. Personality traits were mostly associated with functional connections that were inconsistently present across participants. This suggests that although a fundamental, core functional architecture is preserved across individuals, variable connections outside of that core encompass the inter-individual differences in personality that motivate diverse responses.

## Introduction

Despite the varied and dynamic nature of human environments, the patterns of behavior and cognition that constitute personality tend to be enduring and broadly predictable. A fundamental challenge to neuroscience is uncovering how personality is encoded in the brain [1], [2].

The predominant approach to dimensionalizing personality traits [2], [3] assesses five domains: Neuroticism, Extraversion, Openness to Experience, Agreeableness and Conscientiousness [4], [5]. Studies of the neurobiological substrates of personality traits have largely focused on the most long-standing domains: Neuroticism and Extraversion [2], [6]. The unevenness of coverage of the five principal personality domains is partly ascribable to the constraints inherent in task-based imaging approaches, which require effective cognitive, behavioral or emotional probes that target specific psychological constructs. Consequentially, task-based studies are limited in the breadth of neural systems and cognitive-behavioral constructs that can be effectively probed in a given experiment. Investigating the relationship between personality and brain structure is one method for simultaneously delineating brain systems potentially relevant to all five trait domains [7], but interpretations of structure-behavior relationships remain ambiguous.

Here, we use resting-state functional connectivity (RSFC) analyses to directly examine the brain's functional architecture [8], [9] in relation to each of the five-factor personality traits quantified by the NEO Personality Inventory-Revised (NEO PI-R; [4]). RSFC offers a means to characterize inter-individual differences in intrinsic brain activity while avoiding the constraints of task-based approaches. Recent work has successfully related inter-individual differences in trait measures—such as social competence [10], risk-taking [11], working memory [12], episodic memory [13], aggression [14] and cognitive efficiency [15]—to patterns of RSFC. Much like personality traits, patterns of RSFC observed in these studies are stable across time [5], [16], [17], [18], [19]. In addition, these networks are strikingly similar to the networks activated by a broad spectrum of cognitive-behavioral tasks [20]. In fact, coordinated brain activity at rest has been shown to predict task-evoked activity and behavior [21], [22]. Together these studies suggest that the circuits revealed by analyses of RSFC represent intrinsically organized functional brain networks [23] that persist across tasks, and which appear to serve as the neural foundation on which task-evoked activity, and therefore behavior, is based.

Accordingly, we employed RSFC analyses to identify potentially dissociable intrinsic functional networks associated with each of the five domains of personality quantified by the NEO PI-R: Neuroticism, Extraversion, Openness to Experience, Agreeableness, and Conscientiousness. We chose to examine RSFC with respect to two functionally heterogeneous brain areas involved in diverse aspects of cognition—such as integration of multidimensional information and higher-order executive control—that are commonly investigated in RSFC studies: the anterior cingulate cortex [24], [25] and the precuneus [26]. These regions are thought to be cortical “hubs” with connections spanning the majority of the brain [27], [28], [29]. Based on the neuroimaging literature on personality, we hypothesized that inter-individual variations in personality measures would predict RSFC between our chosen regions of interest and regions implicated in cognitive functions related to each trait. Specifically, we expected that Neuroticism would predict connectivity with regions involved in self- and other-evaluation, such as the dorsomedial prefrontal cortex [7]; Extraversion would predict connectivity with regions implicated in reward and motivation, including the orbitofrontal cortex, insula and the amygdala [7], [30]; Openness to Experience would predict connectivity with regions involved in cognitive flexibility, such as the anterior cingulate cortex [31] and dorsolateral prefrontal cortex [32]; Agreeableness would predict connectivity with regions subserving altruism and social information processing, including the occipital cortex and posterior temporal cortex [33]; and Conscientiousness would predict connectivity with regions involved in planning and self-discipline, such as the lateral prefrontal cortex and medial temporal lobe [2], [7]. Additionally, since the five personality domains have been shown to be relatively independent and to describe non-overlapping traits [4], we expected to observe unique neural correlates for each domain.

## Materials and Methods

### Participants

Resting-state scans were acquired for 39 right-handed adults (18 males, mean age 30±8 years) who completed the NEO Personality Inventory-Revised (NEO PI-R; [4]). The NEO PI-R was designed to measure normal variations of personality in terms of five stable, heritable [34] domains, and it possesses strong reliability and validity [3], [4], [35], [36]. Each participant completed between 1 to 5 resting-state fMRI scans. The first scan session (Scan 1) took place 5–16 months prior to a second session during which two additional resting-state scans were acquired ∼45 minutes apart (Scans 2 and 3). A small number of participants attended a third scanning session 1–2 weeks later, during which two further resting-state scans were acquired ∼45 minutes apart (Scans 4 and 5). For each subject, functional connectivity maps of all scans with less than 3 mm maximum head displacement were averaged to derive the best estimate of that individual's RSFC. Importantly, the number of resting state scans in each participant's RSFC estimates was included as a nuisance covariate for all group-level analyses to avoid introduction of a possible confound. In addition, group-level connectivity maps obtained when all available scans for a subject were used to assess RSFC measures were highly similar to those maps derived from a single resting scan, and both maps showed high Kendall's W concordance as shown in Supporting Figure S1. As expected, the results were more robust when all available scans were included, owing to the fact that the inclusion of multiple scans for a given subject improves our estimate of that subject's RSFC (Supporting Figure S1).

In total, data from five resting-state scans (Scans 1–5) were available for eight participants, data from four resting-state scans (Scans 1–4) were available for one participant, data from three resting-state scans (Scans 1, 2 and 3) were available for six participants, from two scans five months apart (Scans 1 and 2 or 3) for four participants, from two scans 45 minutes apart (Scans 2 and 3) for two participants, and from one scan only (Scan 1 or 2) for 18 participants. Fifteen of these scans were eliminated due to motion as above: five scans from session 1, two from session 2, six from session 3, zero from session 4, and two scans from session 5. Following completion of all scan sessions, participants were asked to return for an additional visit to complete the NEO PI-R. These visits were scheduled at the participants' convenience, and all occurred within one year of each participant's final scan session.

Participants had no history of psychiatric or neurological illness as confirmed by psychiatric clinical assessment. Signed informed consent was obtained prior to participation, and this study was approved by the institutional review boards of New York University (NYU) and the NYU Langone School of Medicine. Data from Scans 1–3 have been reported in several previous studies [10], [17], [18], [21], [24], [37] and are publically available for download at http://fcon_1000.projects.nitrc.org/.

### Assessment

The NEO PI-R was designed by Costa and McCrae [4] (supplanting the original 1985 version). The NEO PI-R form S (self-report) consists of 240 questions answered on a 5-point scale. These questions measure personality across five domains: Neuroticism, Extraversion, Openness to Experience, Agreeableness and Conscientiousness. Each domain is subdivided into six facets, and is intended to be orthogonal to all other domains. Examples of questions include “I can handle myself pretty well in a crisis,” (domain: Neuroticism, facet: Vulnerability) and “I enjoy parties with lots of people” (domain: Extraversion, facet: Gregariousness).

### Data acquisition

For each participant, 6.5-minute resting state functional MRI scans were collected on a 3.0 Tesla Siemens Allegra MRI scanner (197 EPI volumes; TR = 2000 ms; TE = 25 ms; flip angle = 90°; 39 slices; matrix = 64×64; FOV = 192 mm; acquisition voxel size = 3×3×3 mm). During each scan, participants were instructed to rest with their eyes open while the word “Relax” was projected onto the center of the display screen. A high-resolution T1-weighted anatomical image was also acquired using a magnetization prepared gradient echo sequence (MPRAGE, TR = 2500 ms; TE = 4.35 ms; TI = 900 ms; flip angle = 8; 176 slices; FOV = 256 mm).

### Image preprocessing

As detailed in our prior studies [22], [38], data were processed using both AFNI (http://afni.nimh.nih.gov/afni) and FSL (http://www.fmrib.ox.ac.uk). Specific commands can be found in the preprocessing scripts available for download at http://fcon_1000.projects.nitrc.org/. Preprocessing using AFNI consisted of 1) slice time correction for interleaved acquisitions using Fourier interpolation, 2) motion correction using least squares alignment of each volume to the eighth image using Fourier interpolation, 3) despiking of extreme time series outliers using a continuous transformation function, 4) temporal band-pass filtering between 0.009–0.1 Hz using Fourier transformation, and 5) removal of linear and quadratic trends. Additional preprocessing using FSL consisted of 1) spatial smoothing (Gaussian kernel FWHM = 6 mm), and 2) mean-based intensity normalization of all volumes by the same factor (10,000). Next, each participant's preprocessed volume was regressed on nine nuisance signals (global mean, white matter, and CSF signals and six motion parameters). The output of these preprocessing steps was a 4D residual functional volume in each participant's native functional space.

Transformations from native functional and structural space to the Montreal Neurological Institute MNI152 template with 2×2×2 mm resolution were computed using FLIRT and FNIRT [39]. Each participant's high-resolution structural image was registered to the MNI152 template by computing a 12-degree-of-freedom linear affine transformation that was further refined using FNIRT nonlinear registration. Registration of each participant's functional data to their high-resolution structural image was carried out using a linear transformation with 6 degrees of freedom. The structural-to-standard nonlinear warp parameters were then applied to obtain a functional volume in MNI152 standard space.

### Nuisance signal regression

Consistent with common practice in the resting-state fMRI literature, nuisance signals were removed from the data via multiple regression before functional connectivity analyses were performed. This step is designed to control for the effects of physiological processes, such as fluctuations related to motion and cardiac and respiratory cycles [40]. Specifically, each individual's 4D time series data were regressed on nine predictors: white matter (WM), cerebrospinal fluid (CSF), the global signal, and six motion parameters. The global signal regressor was generated by averaging across the time series of all voxels in the brain mask. The WM and CSF covariates were generated by segmenting each individual's high-resolution structural image (using FAST in FSL). The resulting segmented WM and CSF images were thresholded to ensure 80% tissue type probability. These thresholded masks were then applied to each individual's time series, and a mean time series was calculated by averaging across time series of all voxels within each mask. The six motion parameters were calculated in the motion-correction step during preprocessing. Movement in each of the three cardinal directions (X, Y, and Z) and rotational movement around three axes (pitch, yaw, and roll) were included for each individual.

### Individual seed-based functional connectivity analysis

**Figure 1:**
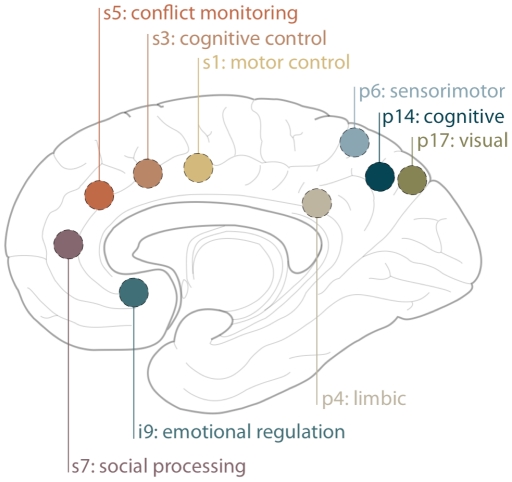
Seed locations. General location of the nine seeds: five within the anterior cingulate cortex (ACC; seeds s1, s3, s5, s7 and i9) and four within the precuneus (PCU; seeds p4, p6, p14, p17). Also shown are associated functions of each of these regions [24], [25], [26]. Seed coordinates are listed in Supporting Table S2.

For seed placement, we selected the anterior cingulate cortex (ACC) and the precuneus (PCU), two functionally heterogeneous brain areas known to be involved in diverse aspects of cognition—such as integration of information and higher-order executive control—and that are commonly investigated in RSFC studies. We created spherical seed regions of interest (diameter = 8 mm) centered at each of these coordinates in both the left and right hemispheres for use in our RSFC analyses: five in the anterior cingulate cortex (ACC; [25]) and four in the precuneus (PCU; [26]). Seed locations are shown in Figure 1 and coordinates are listed in Supporting Table S1. As detailed in prior studies [22], each individual's residual 4D time series data were spatially normalized by applying the previously computed transformation to the MNI152 standard space. Then the time series for each seed was extracted from these data. Time series were averaged across all voxels in each seed region of interest (ROI). For each individual dataset, the correlation between the time series of the seed ROI and that of each voxel in the brain was determined. This analysis was implemented using 3dfim+ in AFNI to produce individual-level correlation maps of all voxels that were positively or negatively correlated with the seed's time series. Finally, these individual-level correlation maps were converted to Z-value maps using Fisher's r-to-z transformation for subsequent group-level analyses.

### Group-level analyses

Group-level mixed-effects analyses were carried out using ordinary least squares, as implemented in FSL FEAT. Demeaned personality domain scores were included as simultaneous covariates of interest in one model, as well as analyzed independently in separate models. Across all seeds and domains, Kendall's W was calculated between the models in which the domains were included simultaneously and the models in which they were included separately to determine the correspondence between the two types of group-level modeling (Supporting Figure S2). We chose to focus on the results of the model in which all five personality domain scores were included as simultaneous covariates of interest, because that model design reveals the associations between RSFC and personality that are unique to each personality domain. Correlations between personality domain scores and the number of resting-state scans obtained per participant—that were included in the final analysis—were negligible (ranging from r = 0.019 for Neuroticism to r = −0.303 for Agreeableness). Nevertheless, we covaried the number of resting state scans included per participant to minimize artifactual contributions. Nuisance covariates for age and sex were included as well. Gaussian random field theory was used to correct for multiple comparisons at the cluster-level (Z>2.3; p<0.05, corrected).

For each seed region, group-level analyses produced the following two types of thresholded z-statistic maps: 1) maps of voxels exhibiting significant positive and negative functional connectivity with the seed across all individuals and 2) maps of voxels whose positive or negative functional connectivity with the seed exhibited significant variation in association with the personality domain scores (i.e., regions in which connectivity with the seed was predicted by score). Regions whose RSFC with the relevant seed ROI exhibited a significant relationship with personality scores were sorted according to the valence of their RSFC—that is, whether the region was significantly (i.e., consistently) positively correlated (“invariant positive”), significantly negatively correlated (“invariant negative”), or not significantly correlated (“variable”) with the relevant seed ROI, across participants. Finally, we conducted conjunction analyses to quantify the number of voxels exhibiting relationships with all five personality domains. This was accomplished by binarizing group-level thresholded maps of positive, negative and variable RSFC across all seeds and then summing them to create a conjunction map. The resultant map was then thresholded to identify areas that were common or unique to all RSFC maps.

### Confirmatory analyses for personality-RSFC relationships

To ensure the robustness of our findings relating personality scores to specific functional connections, we verified our findings using both a split-half analysis and non-parametric testing. Specifically, we 1) randomly split the sample into two halves, and 2) for each functional connection identified as having a significant relationship with personality score in the entire sample (i.e., in the primary analyses), we verified the presence of the RSFC/personality score relationship in each of the two split groups. This analysis minimizes the likelihood that the observed relationships were driven by a subset of participants. Then, in a separate confirmatory analysis, we employed non-parametric testing to verify the presence of significant RSFC/personality score relationships emerging from our primary analyses. Specifically, for each of the significant functional connection/personality score relationships identified in the primary analysis, we 1) generated 5000 random pairings between the strength of RSFC for that specific connection and personality scores across subjects, allowing us to build a null distribution of r-values, and 2) calculated the p-value for each relationship examined based upon where the true RSFC/personality score correlation fell within that null distribution (e.g., if the true r-value ranked in the 97.5^th^ percentile of the distribution, the p-value would be 0.025 [1−.975]). The confirmatory nature of this analysis justified forgoing corrections for multiple comparisons. Results for both split-half and non-parametric confirmatory analyses are shown in Supporting Table S1.

## Results

### Personality domain scores

In our sample, participant scores on Neuroticism (N; mean ± SD: 78±28; range: 12–142), Extraversion (E; 119±20; range: 79–168), Openness to Experience (O; 128±21; range: 92–166), Agreeableness (A; 125±15; range: 88–151), and Conscientiousness (C; 122±22; range: 70–176) closely matched population norms (N: 79±21; E: 109±18; O: 111±17; A: 124±16; C: 123±18) [4]. Between-domain score correlations are shown in Supporting Figure S3.

### Personality domain scores predicted RSFC

**Figure 2:**
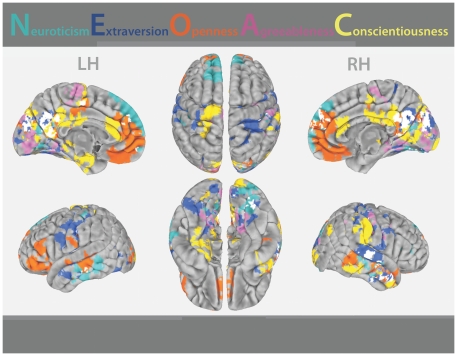
Personality trait measures predicted RSFC. Connections identified as having a relationship with personality, grouped by color according to the personality domain that predicted their RSFC. For the purpose of illustration, significant findings were collapsed across seed regions and RSFC/personality score relationship valence (i.e., whether the correlation was significantly positive or negative). Individual seed region findings are presented separately in Figures 3–5. White represents overlap of findings for multiple (one or more) personality domains predicting RSFC. LH = left hemisphere; RH = right hemisphere.

**Figure 3:**
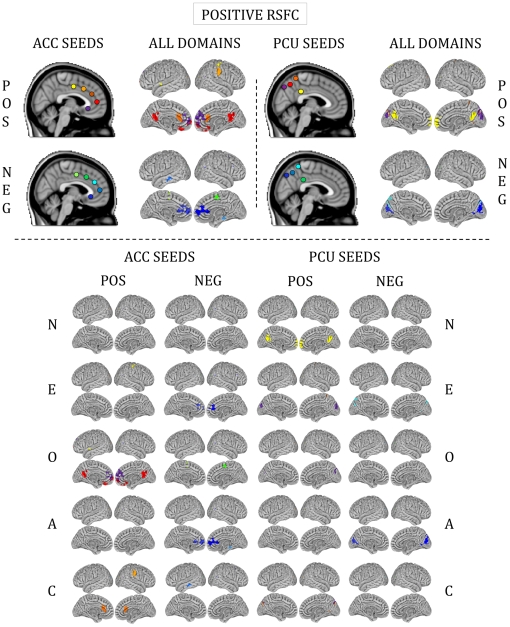
Functional connections predicted by positive RSFC. Surface maps of regions whose RSFC with ACC and PCU seeds was predicted by personality. These maps illustrate positive RSFC only. Colors on the surface maps represent RSFC with the seeds shown at the top in corresponding colors. “All domains” refers to all five personality domains combined into a single map. POS = positive relationships (stronger RSFC relationships with higher personality score); NEG = negative relationships (stronger RSFC relationships with lower personality score). N = Neuroticism; E = Extraversion; O = Openness to Experience; A = Agreeableness; C = Conscientiousness.

**Figure 4 pone-0027633-g004:**
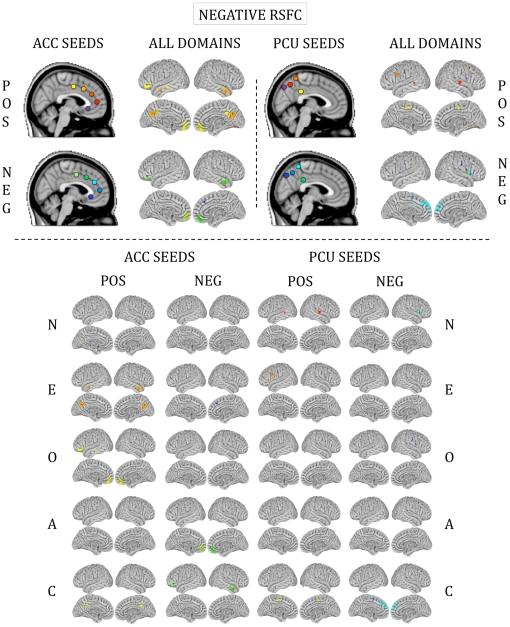
Functional connections predicted by negative RSFC. Surface maps of regions whose RSFC with ACC and PCU seeds was predicted by personality. These maps illustrate negative RSFC only. Colors on the surface maps represent RSFC with the seeds shown at the top in corresponding colors. “All domains” refers to all five personality domains combined into a single map. POS = positive relationships (stronger RSFC relationships with higher personality score); NEG = negative relationships (stronger RSFC relationships with lower personality score). N = Neuroticism; E = Extraversion; O = Openness to Experience; A = Agreeableness; C = Conscientiousness.

**Figure 5:**
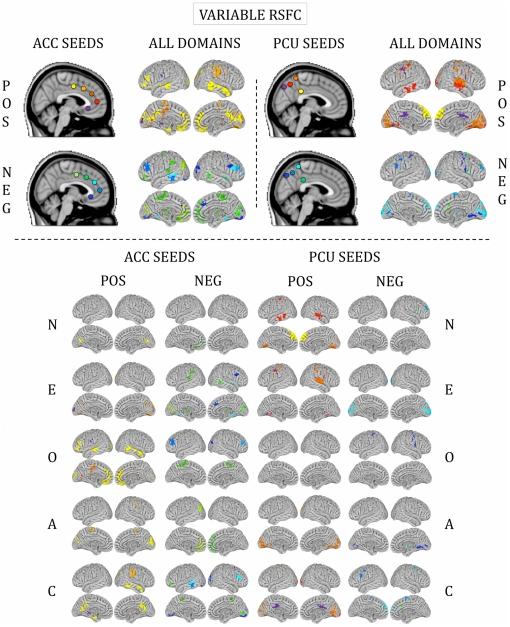
Functional connections predicted by variable RSFC. Surface maps of regions whose RSFC with ACC and PCU seeds was predicted by personality. These maps illustrate variable RSFC only. Colors on the surface maps represent RSFC with the seeds shown at the top in corresponding colors. “All domains” refers to all five personality domains combined into a single map. POS = positive relationships (stronger RSFC relationships with higher personality score); NEG = negative relationships (stronger RSFC relationships with lower personality score). N = Neuroticism; E = Extraversion; O = Openness to Experience; A = Agreeableness; C = Conscientiousness.

For all five personality domains, we detected significant RSFC-personality relationships between our *a priori* seeds (Figure 1) and expected cognitive and affective processing regions (Figures 2, 3, 4 and 5). The majority of regions whose RSFC with the ACC seeds (Figure 1) was predicted by personality were located in the medial prefrontal cortex, paracingulate gyrus and anterior/central precuneus (Figures 3, 4 and 5). The majority of regions whose RSFC with the PCU seeds (Figure 1) was predicted by personality were located in and surrounding the precuneus, the dorsomedial prefrontal cortex and the posterior cingulate gyrus, as well as the primary motor and visual cortices (Figures 3, 4 and 5). For example, Neuroticism scores predicted positive RSFC between PCU seed p4 (Figure 1) and the central precuneus and dorsomedial prefrontal cortex (Figure 3). Regions whose RSFC with ACC seeds (all except ACC s5; Figure 1) was invariantly positive were located at long distances from the seed region of interest or were located in distinct functional areas (i.e., the connections were long-range; Figure 3). By contrast, regions whose RSFC with PCU seeds was invariably positive were primarily located proximal to the seed region of interest or were located in the same anatomical or functional region (i.e., the connections were local; Figure 3). For ACC seeds, this pattern was also evident for RSFC that was both invariantly negative and variably present across participants (Figures 4, 5). The above results are summarized in Supporting Table S2, and surface maps of all personality-RSFC relationships can be seen in Figures 3, 4 and 5. Supplementary single-scan, split-half and non-parametric analyses confirmed the robustness of our results, as shown in Supporting Figure S1 and Supporting Table S1.

### Brain regions whose RSFC was predicted by personality did not overlap

Using a voxel-wise conjunction analysis, we determined that there were no voxels common to all five personality-RSFC relationships. With minor exceptions (the white areas in Figure 2 illustrate voxels common to more than one but fewer than five domains), each domain of personality predicted RSFC between seed ROIs (ACC and PCU; Figure 1) and unique sets of brain regions (Figure 2). Across all domains, the pattern of regions whose connectivity was predicted by personality corresponded with functional subsystems in the brain, particularly default-mode network fractionations (Figure 2; [18], [41]). For example, Neuroticism predicted RSFC in the dorsomedial prefrontal cortex subsystem known to be involved in self-referential processing [41] and emotional regulation [7], [42]. Functional connections identified as having a significant relationship with Neuroticism were also located in the middle temporal gyrus and temporal pole, consistent with activation studies of this trait during fearful anticipation and negative emotions [43], [44]. Extraversion predicted RSFC in the lateral paralimbic group implicated in motivation and reward [33], [42], [45], [46]. Functional connections identified as having a significant relationship with Extraversion were also located in the fusiform gyrus, consistent with prior studies [47] and the area's role in social attention and face recognition [48]. Openness to Experience predicted RSFC with the midline core “hubs” of the default mode network, known to be involved in integration of the self and the environment [31], [41]. Functional connections identified as having a significant relationship with Openness to Experience were also located in the dorsolateral prefrontal cortex, a region associated with working memory, intelligence, creativity and the intellect facet of Openness to Experience [31], [32], [49]. Agreeableness predicted RSFC with posteromedial extrastriate regions as well as some primary sensorimotor areas, the combination of which is reported to be involved in social and emotional attention [7], [33]. Finally, Conscientiousness predicted RSFC with the medial temporal lobe subsystem involved in future-oriented episodic judgment and planning [41]. Although our model design was organized to maximize independence among the domain-RSFC patterns, this independence persisted when the five personality domains were analyzed in separate models. This is demonstrated by the high Kendall's W concordance across all seeds and domains between 1) maps generated by the group analysis model where all five domains were analyzed simultaneously, and 2) maps generated when all five domains were analyzed independently in separate group analysis models (Supporting Figure S2).

### Unpredicted personality-RSFC relationships

Unexpected relationships also emerged: all five domains except Openness to Experience predicted RSFC between ACC seeds and the cerebellar vermis. Openness to Experience predicted RSFC between PCU seeds and the right cerebellar hemisphere. Additionally, all five domains predicted RSFC between at least one seed and the visual cortex (Figures 3, 4 and 5). All relationships are listed in Supporting Table S2.

### Functional connections predicted by personality were inconsistently present across participants

**Figure 6:**
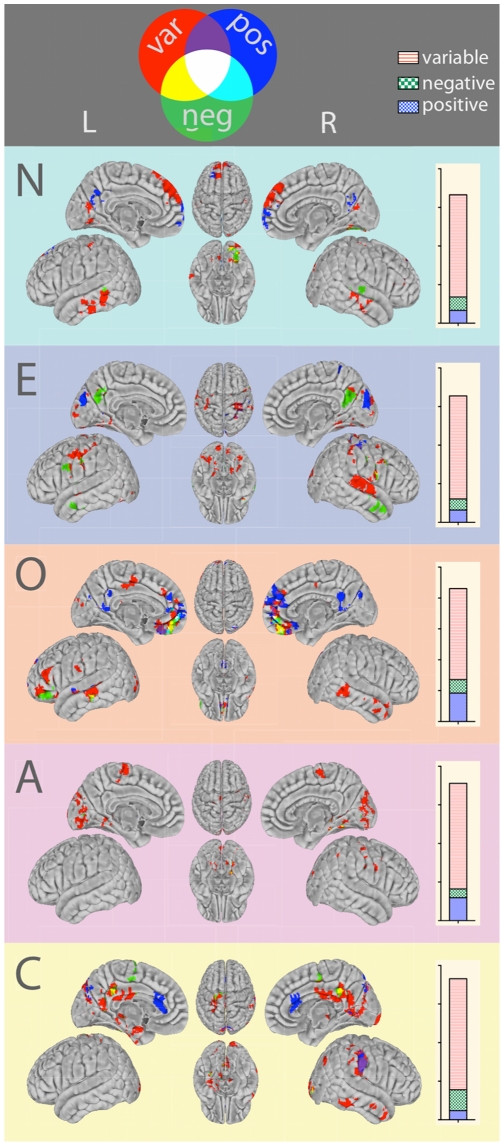
Functional connections predicted by personality are variable across participants. Regions whose RSFC with ACC and PCU seeds was predicted by each of the five personality domains, grouped by color according to the valence of their RSFC—that is, whether the region was significantly (i.e., consistently) positively correlated (“invariant positive”; pos), significantly negatively correlated (“invariant negative”; neg), or not significantly correlated (“variable”; var) with the relevant seed region of interest, across all ACC and PCU seeds. Results are segregated by the relevant personality domain that predicted RSFC. Histograms to the right of the surface maps quantify the number of voxels identified as having a relationship with personality score, grouped according to their RSFC valence. As illustrated at the top of the figure, for both brain regions and histogram categories, blue corresponds to “positive” RSFC valence, green corresponds to “negative” RSFC valence, and red corresponds to “variable” RSFC valence. L = left hemisphere; R = right hemisphere; N = Neuroticism; E = Extraversion; O = Openness to Experience; A = Agreeableness; C = Conscientiousness.

Across all personality domains, the majority of functional connections found to be related to personality scores were variably present across participants (Figure 6). In other words, these connections did not exhibit statistically significant positive or negative RSFC with the seed regions across the sample. Of note, these connections were frequently located on the boundaries of regions whose RSFC with relevant seed ROIs was consistently significantly positive or negative across the sample.

## Discussion

Unique patterns in the brain's intrinsic functional architecture reflected each of the five personality domains assessed by the NEO PI-R. RSFC patterns of the anterior cingulate cortex (ACC) and precuneus (PCU), regions commonly implicated in the regulation and integration of higher order information [24], [25], [26], [27], [28], [29], were correlated with personality domain scores. These results highlight the utility of examining the brain's intrinsic functional architecture to identify neural markers of complex traits, such as in the study of psychiatric or personality disorders.

### Personality-RSFC network functions matched psychological qualities associated with each trait

Cognitive and psychological functions associated with the regions whose RSFC with ACC and PCU seeds was predicted by personality were consistent with known qualities about each relevant personality domain [7], [35]. For instance, Neuroticism predicted RSFC with brain areas involved in self-evaluation and fear [41], [43], [44], and is known to be associated with anxiety and self-consciousness [35], [50]. Extraversion predicted RSFC with brain areas involved in reward and motivation [33], [42], [45], [46], and is implicated in gregariousness and excitement-seeking [35], [51]. Openness to Experience predicted RSFC with brain areas involved in cognitive flexibility and imagination [31], [32], [41], and is associated with fantasy, intellectual curiosity and exploration [2], [35]. Agreeableness predicted RSFC with brain areas involved in empathy and social information processing [7], [33], and is linked with compassion and friendliness [35]. Finally, Conscientiousness predicted RSFC with brain areas involved in planning and self-discipline [41], and is implicated in carefulness, industriousness and organization [35], [52].

### Personality relates to a network of functional connections

Our RSFC findings extend prior studies that linked personality traits to regional differences in brain structure [7], [53], [54], [55] or function [56], [57], [58], [59], [60], [61]. By contrast, the present findings emphasize the importance of considering functional relationships *between* regions in order to map complex brain-behavior relationships, rather than being limited to volumetric differences or the momentary responsivity of individual brain regions or sets of regions.

For example, Neuroticism predicted positive RSFC between PCU seed p4—a region involved in limbic processing (Figure 1; [26])—and the surrounding precuneus (Figure 3). This region of the precuneus is implicated in social [62] and emotional [63] functions, especially among individuals high in Neuroticism [44], [64], [65], [66], who tend to be more socially dysfunctional [67] and reactive to negative emotional experiences [35]. Yet the precuneus is a large, functionally heterogeneous region [26], and it cannot be assumed to be solely responsible for Neuroticism. Instead, it is only when we consider the functional relationship *between* the seed and additional regions that we can interpret how these areas interact in unique ways to produce a framework for modulating behavioral responses to environmental stimuli.

In this example, individuals scoring high on Neuroticism exhibit a more tightly connected “limbic” precuneus, as reflected in the increased local connectivity in this area (Figure 3). But these individuals also demonstrated increased connectivity with the central “cognitive” precuneus (Figure 3; [26]). As the seed region (p4; Figure 1) is involved in limbic processing [26] and the central precuneus is involved in higher-order cognitive function [68], [69], [70], this connection suggests that Neuroticism involves increased integration of social and emotional information [7], [35], [71] and may relate to increased sensitivity to social-emotional cognitive conflicts [72], [73]. Yet this same seed (p4; Figure 1) is also connected to the dorsomedial prefrontal cortex (Figure 3), a region involved in self-evaluation [41] and social interaction [74]. A relationship between these cognitive processes (i.e., self-evaluation and integration of social and emotional information) is highly consistent with the psychological qualities inherent to this personality trait [35]. Moreover, the distributed pattern of connectivity shown here between seed p4 (Figure 1), multiple regions of the precuneus, and the dorsomedial prefrontal cortex implies the existence of a network of inter-related regions underlying Neuroticism that are each individually identified by task-based studies, but captured entirely by RSFC. The networks of interconnected brain regions delineated by RSFC studies would otherwise be appreciated piecemeal through the lens of specific task contrasts [75]. Thus, resting-state fMRI is well-suited to address complex constructs such as personality. These results set the stage for complementary task-based studies that can be particularly useful in parsing and supporting the interpretation of resting-state fMRI results.

### Contributions of unpredicted brain regions to personality

We also detected less intuitive results. For example, all five personality domains predicted RSFC between numerous seeds and primary motor and sensory regions (e.g., occipital cortex; Figures 3, 4 and 5). Task-based studies have also found relationships between the occipital cortex and higher-order behavioral traits, such as word [76] and food picture [77] recognition, risky decision-making [78] and auditory expectation [79]. Typically these findings are attributed to the visual components inherent to the task paradigms employed in the particular study. But Kober et al. [33] suggested that visual cortex activity may contribute to attentional processing of emotionally-valenced stimuli, rather than being limited to low-level sensory processing. As some of these seeds (e.g., seed s1 and Openness to Experience; Figures 1 and 5) also demonstrated a connection with prefrontal regions mediating higher-order cognitive function, this suggests that dynamic interactions of large-scale networks including low-level sensory and high-order cognitive brain regions subserve complex thoughts and behavior [80].

Of particular interest is the ubiquitous relationship demonstrated between personality domain scores and the cerebellum, especially the cerebellar vermis. Previous studies implicated the cerebellum in non-motor [81], higher cognitive functions [82], [83], and cerebellar lesions have been shown to produce personality changes [84], [85]. This suggests that full coverage of the cerebellum should be a priority in future neuroimaging studies of personality.

### Significance of variable RSFC

In analyses of RSFC data from over 1000 participants, a “core” intrinsic, functional architecture was found to be consistent across individuals and imaging centers [38], [86]. However, despite striking similarities, substantial inter-individual variations could also be appreciated. In a previous study, we found that autistic traits were related to functional connections that were variably present across participants (as opposed to relatively invariant positive or negative) [10]. Here, we also found that the majority of functional connections exhibiting relationships with personality had inconsistent patterns of connectivity across participants (Figure 6). This suggests that although a fundamental, core functional architecture is preserved across individuals, variable connections outside of that core may underlie the inter-individual differences in personality that motivate diverse responses.

### Clinical implications

Prior efforts to use behavior-RSFC relationships to target clinically-relevant neural circuits [10], [11], [12], [13], [14], [15] are continued in the present study, as differences in five-factor personality scores have been linked to a range of clinical pathology including personality disorders [87], [88], mood and anxiety disorders [89], [90] and attention-deficit/hyperactivity disorder [91]. As our knowledge of the neural circuitry of personality improves, so will too our ability to identify abnormalities in these personality networks relevant to neuropsychopathology. Future investigations of the neural circuits identified by these studies will enhance our understanding of their functional significance, ultimately improving our ability to diagnose and treat a wide range of neuropsychiatric disorders.

### Limitations

The scope of the present work is limited to the identification of markers of inter-individual variation in personality traits within the brain's intrinsic functional architecture. While our findings hold great potential for the identification of markers related to personality pathology, their correlational nature constrains the interpretations they can support. Specifically, we cannot yet conclude that our results represent the cortical embodiment of inter-individual differences in personality traits nor the mechanisms through which such differences manifest in individuals.

An important methodological limitation of seed-based RSFC studies, including ours, is the *a priori* selection of regions of interest. A potential strategy would have been to select regions of interest based on previous neuroimaging studies of personality [7], [43], [54], [73], [92]. However, the sheer number of published results and the diversity of task paradigms employed across studies did not make this a practical option. Instead, we focused on the brain's cortical “hubs”: regions that are widely connected with the rest of the brain and are of central importance to diverse cognitive, affective, and motivational processes [27], [28], [29]. By examining the relationship of these hubs with the areas to which they are functionally connected, this approach emphasizes the degree to which personality traits are associated with unique distributed networks of regions, rather than being localized in a few specific regions. Future work using RSFC can systematically interrogate other regions and networks such as the striatum [93], [94], amygdala [95], [96], [97] and insula [10], [98], [99] to gain further insights into the functional specialization of personality traits.

Our study was also limited by its modest sample size. An exhaustive mapping of RSFC/personality relationships in the functional connectome would require levels of statistical correction beyond what is practical for the present sample. Though less comprehensive, seed-based correlation analysis limits the scope of statistical interrogation, making exploration of modestly powered samples feasible. Future studies incorporating large-scale, data-driven methods [100] are needed to examine the neural correlates of personality more comprehensively.

### Conclusion

Consistent with the neural network model of personality [101], our results suggest that distinct personality domains are encoded by dissociable patterns of functional connectivity among specific brain regions, despite the presence of modest inter-domain score correlations (Supporting Figure S3). Further appreciation of how personality is encoded within RSFC patterns will integrate with previous multimodal approaches [7], [100], [102] and inform future studies of personality, mood and anxiety disorders, and their development over the first several decades of life.

## Supporting Information

Figure S1 **Comparison of results using single- and multiple-scan session data.** Comparison of surface maps of regions whose RSFC with PCU seeds was predicted by personality between a representative single-scan analysis (i.e., data from Scan 1 only; SINGLE) and the analysis used in the main text (i.e., data averaged across all scan sessions; MULTIPLE). Maps are not sorted according to their RSFC valence (i.e., positive, negative, or variable). Kendall's W concordance between the SINGLE and MULTIPLE maps for both positive (i.e., stronger RSFC relationships with higher personality score; POS) and negative (i.e., stronger RSFC relationships with lower personality score; NEG) behavior relationships is listed in the third column. Colors are consistent with labeling in Figure 3, 4 and 5.
(TIF)

Figure S2 **Concordance between models in which personality domains are included simultaneously and models in which domains are analyzed separately.** Kendall's W comparison between group maps when all five personality domains are included simultaneously in the group analysis model, and when all five personality domains are analyzed in separate models. Kendall's W concordance between these two models was calculated for every seed (Y axis) and every personality domain (X axis). Seed locations are shown in Figure 1 and coordinates are listed in Supporting Table S2. N = Neuroticism; E = Extraversion; O = Openness to Experience; A = Agreeableness; C = Conscientiousness.
(EPS)

Figure S3 **Correlations between personality domain scores across subjects.** Correlations between all possible pairs of personality domain scores across all subjects. Numeric values are shown in the upper triangle; corresponding colors are shown in the lower triangle. Red colors represent positive correlations; blue colors represent negative correlations. Darker colors correspond to higher absolute values. N = Neuroticism; E = Extraversion; O = Openness to Experience; A = Agreeableness; C = Conscientiousness.
(TIF)

Table S1 **Confirmatory split-half and non-parametric analyses.** Table of results from confirmatory split-half and non-parametric analyses. Results are only shown for seeds demonstrating significant RSFC relationships predicted by personality. Results are listed for each personality domain and for both positive (i.e., stronger RSFC relationships with higher personality score; POS) and negative (i.e., stronger RSFC relationships with lower personality score; NEG) behavior relationships. Results include: 1) for the split-half analysis, r-values for the full sample and both sample halves, and 2) for the non-parametric analysis, p-values. The two P-values greater than 0.05 (i.e., those connections for which the true RSFC/personality score correlation as determined our primary analysis *did not* fall outside of the 95% confidence interval of the null distribution of r-values) are highlighted in yellow. Seed labels are consistent with Figure 1. R = right-sided seed; L = left-sided seed.
(XLS)

Table S2 **Details of all functional connections predicted by personality.** Table of local peaks associated with each RSFC relationship for each domain of personality. Coordinates are in standard MNI152 space. Seed locations are shown in Figure 1. Behavior refers to behavioral relationships: positive relationships are stronger with higher personality domain scores, negative relationships are stronger with lower personality domain scores. pos = positive relationship; neg = negative relationship. N = Neuroticism; E = Extraversion; O = Openness; A = Agreeableness; C = Conscientiousness.
(DOC)
