# Supplementary figures and images for: Personality Is Reflected in the Brain's Intrinsic Functional Architecture
Source: PLoS One. 2011 Nov 30;6(11):e27633. doi: 10.1371/journal.pone.0027633 (PMC3227579; doi:10.1371/journal.pone.0027633)

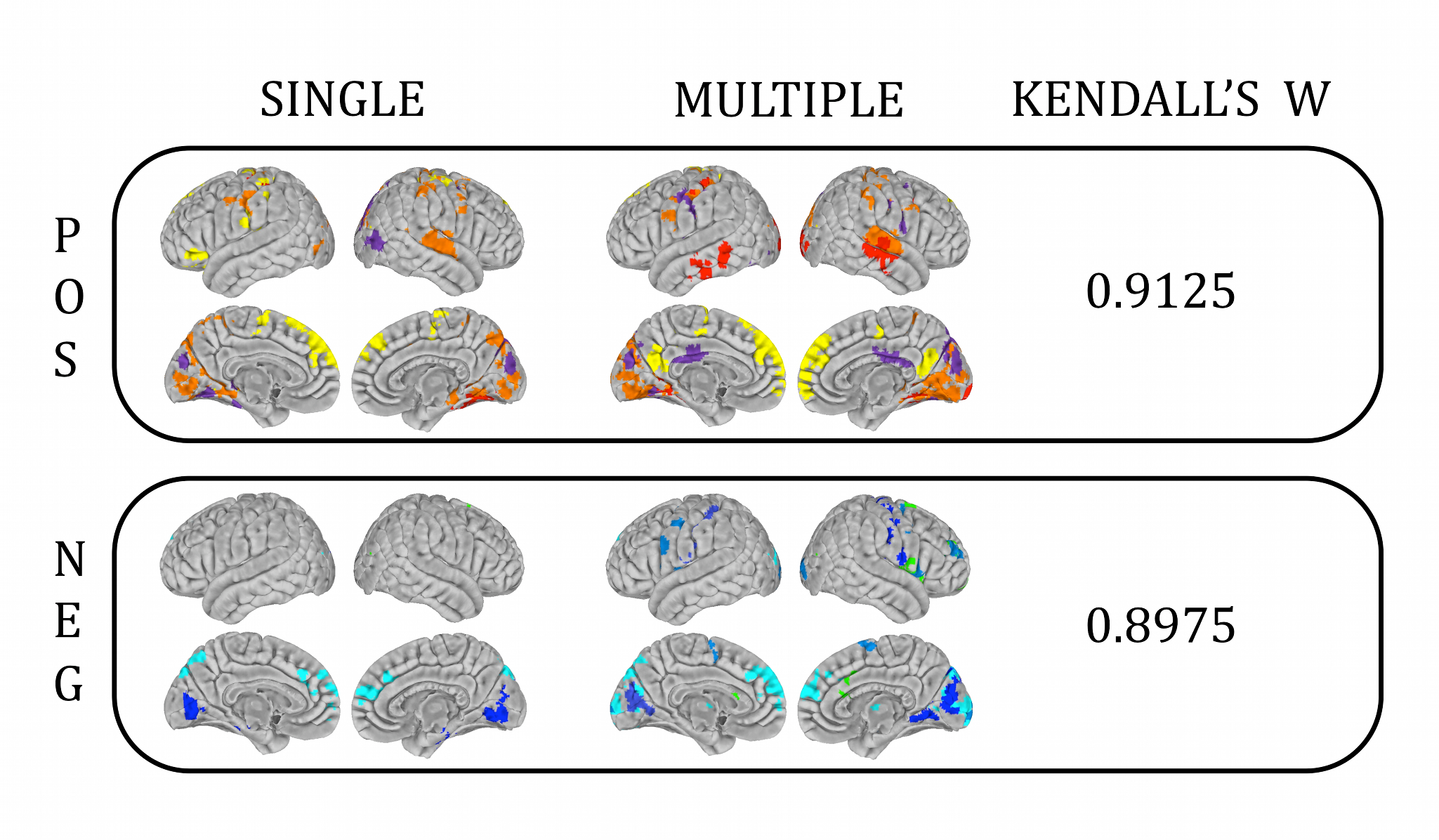

Supplement: Figure S1 — Comparison of results using single- and multiple-scan session data. Comparison of surface maps of regions whose RSFC with PCU seeds was predicted by personality between a representative single-scan analysis (i.e., data from Scan 1 only; SINGLE) and the analysis used in the main text (i.e., data averaged across all scan sessions; MULTIPLE). Maps are not sorted according to their RSFC valence (i.e., positive, negative, or variable). Kendall's W concordance between the SINGLE and MULTIPLE maps for both positive (i.e., stronger RSFC relationships with higher personality score; POS) and negative (i.e., stronger RSFC relationships with lower personality score; NEG) behavior relationships is listed in the third column. Colors are consistent with labeling in Figure 3, 4 and 5. (TIF) [file pone.0027633.s001.tif]

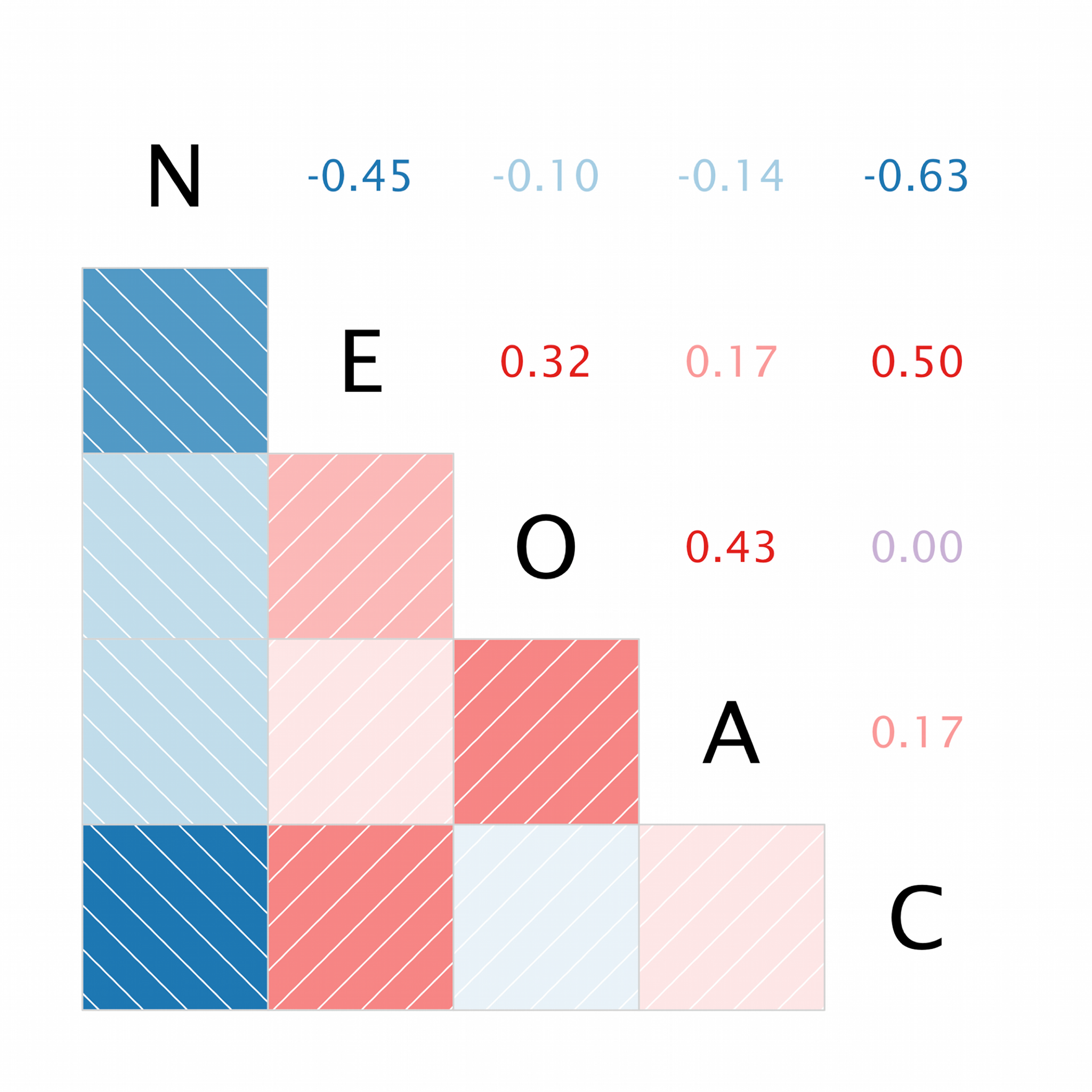

Supplement: Figure S3 — Correlations between personality domain scores across subjects. Correlations between all possible pairs of personality domain scores across all subjects. Numeric values are shown in the upper triangle; corresponding colors are shown in the lower triangle. Red colors represent positive correlations; blue colors represent negative correlations. Darker colors correspond to higher absolute values. N = Neuroticism; E = Extraversion; O = Openness to Experience; A = Agreeableness; C = Conscientiousness. (TIF) [file pone.0027633.s003.tif]
