# Supplementary material for: Personality Is Reflected in the Brain's Intrinsic Functional Architecture
Source: PLoS One. 2011 Nov 30;6(11):e27633. doi: 10.1371/journal.pone.0027633 (PMC3227579; doi:10.1371/journal.pone.0027633)
Supplement: Table S2 — Details of all functional connections predicted by personality. Table of local peaks associated with each RSFC relationship for each domain of personality. Coordinates are in standard MNI152 space. Seed locations are shown in Figure 1. Behavior refers to behavioral relationships: positive relationships are stronger with higher personality domain scores, negative relationships are stronger with lower personality domain scores. pos = positive relationship; neg = negative relationship. N = Neuroticism; E = Extraversion; O = Openness; A = Agreeableness; C = Conscientiousness. (DOC) [file pone.0027633.s005.doc]

| **Domain** | **Seed** | **Coordinate** | **Region** | **x** | **y** | **z** | **Behavior** |
| --- | --- | --- | --- | --- | --- | --- | --- |
| A | i9R | 5, 25, -10 | Right Medial Frontal Gyrus | 6 | 54 | 8 | neg |
|  |  |  | Left Middle Frontal Gyrus | -24 | 46 | -6 | neg |
|  | s1L | -5, -10, 47 | Left Medial Frontal Gyrus | -4 | 56 | 4 | neg |
|  |  |  | Left Angular Gyrus | -52 | -74 | 32 | neg |
|  |  |  | Right Medial Frontal Gyrus | 6 | 50 | 44 | neg |
|  |  |  | Left Rectal Gyrus | 0 | 38 | -22 | neg |
|  | s1R | 5, -10, 47 | Left Angular Gyrus | -46 | -76 | 30 | neg |
|  | s3L | -5, 14, 42 | Right Pyramis | 28 | -56 | -36 | neg |
|  | s7R | 5, 47, 11 | Right Parahippocampal gyrus | 16 | -24 | -14 | neg |
|  | p6L | -2, -47, 58 | Right Cerebellar tonsil | 10 | -38 | -48 | neg |
|  | p6R | 2, -47, 58 | Right Lentiform nucleus | 24 | -2 | 18 | neg |
|  |  |  | Left Thalamus | -8 | -4 | 4 | neg |
|  | p17L | -1, -78, 43 | Left Cuneus | 0 | -80 | 10 | neg |
|  |  |  | Right Precuneus | 12 | -80 | 40 | neg |
|  | p17R | 1, -78, 43 | Left Cuneus | 0 | -80 | 10 | neg |
|  |  |  | Right Culmen | 8 | -44 | -4 | neg |
|  |  |  | Right Precuneus | 12 | -80 | 40 | neg |
|  |  |  | Left Middle Occipital Gyrus | 34 | -74 | 8 | neg |
|  | s1L | -5, -10, 47 | Right Inferior Pareital Lobule | 36 | -50 | 46 | pos |
|  | s1R | 5, -10, 47 | Left Cuneus | -10 | -78 | 10 | pos |
|  |  |  | Right Lingual Gyrus | 28 | -78 | -12 | pos |
|  |  |  | Right Middle Occipital Gyrus | 14 | -102 | 10 | pos |
|  |  |  | Right Precuneus | 6 | -88 | 40 | pos |
|  | s3L | -5, 14, 42 | Right Medial Frontal Gyrus | 4 | -16 | 64 | pos |
|  |  |  | Right Precentral Gyrus | 46 | -18 | 54 | pos |
|  |  |  | Left Postcentral Gyrus | -14 | -42 | 66 | pos |
|  |  |  | Right Precentral Gyrus | 60 | 2 | 28 | pos |
|  | p6L | -2, -47, 58 | Left Middle Occipital Gyrus | -24 | -88 | 18 | pos |
|  |  |  | Right Parahippocampal Gyrus | 22 | -52 | -8 | pos |
|  |  |  | Right Middle Occipital Gyrus | 14 | -92 | 12 | pos |
|  |  |  | Left Lingual Gyrus | -14 | -76 | -8 | pos |
|  |  |  | Left Parahippocampal Gyrus | -26 | -38 | -10 | pos |
|  | p6R | 2, -47, 58 | Right Parahippocampal Gyrus | 22 | -50 | -6 | pos |
|  |  |  | Right Middle Occipital Gyrus | 12 | -92 | 10 | pos |
|  |  |  | Left Middle Occipital Gyrus | 24 | -88 | 18 | pos |
|  |  |  | Left Parahippocampal Gyrus | 26 | -38 | -8 | pos |
|  |  |  | Left Lingual Gyrus | 12 | -66 | -2 | pos |
|  |  |  | Right Precuneus | 22 | -84 | 38 | pos |
|  | p14L | -2, -64, 45 | Right Middle Frontal Gyrus | 46 | 2 | 44 | pos |
|  | p14R | 2, -64, 45 | Right Medial Frontal Gyrus | 46 | 0 | 44 | pos |
| C | i9L | -5, 25, 10 | Left Middle Occipital Gyrus | -40 | -76 | -14 | neg |
|  |  |  | Right Lateral Occipital Cortex | 26 | -72 | 22 | neg |
|  |  |  | Right Lingual Gyrus | 26 | -76 | -16 | neg |
|  |  |  | Left Lingual Gyrus | -8 | -68 | -2 | neg |
|  |  |  | Left Parahippocampal Gyrus | -34 | -44 | -6 | neg |
|  |  |  | Right Precuneus | 14 | -86 | 46 | neg |
|  | i9R | 5, 25, -10 | Right Superior Frontal Gyrus | 18 | 10 | 54 | neg |
|  | s1L | -5, -10, 47 | Right Uvula | 12 | -86 | -32 | neg |
|  |  |  | Left Culmen | -2 | -70 | -6 | neg |
|  |  |  | Right Middle Occipital Gyrus | 42 | -82 | 0 | neg |
|  | s3L | -5, 14, 42 | Right Paracentral Lobule | 10 | -34 | 56 | neg |
|  | s3R | 5, 14, 42 | Right Middle Temporal Gyrus | 46 | -16 | -18 | neg |
|  |  |  | Left Medial Frontal Gyrus | -12 | -24 | 54 | neg |
|  |  |  | Right Postcentral Gyrus | 40 | -28 | 56 | neg |
|  | s5L | -5, 24, 28 | Left Inferior Frontal Gyrus | -34 | 2 | 34 | neg |
|  |  |  | Left Middle Temporal Gyrus | -60 | -50 | 2 | neg |
|  | s5R | 5, 34, 28 | Right Inferior Frontal Gyrus | 58 | 30 | 20 | neg |
|  | s7L | -5, 47, 11 | Left Middle Temporal Gyrus | -58 | -38 | -12 | neg |
|  | s7R | 5, 47, 11 | Left Middle Temporal Gyrus | -50 | -32 | -16 | neg |
|  | p4L | -2, -36, 35 | Right Middle Frontal Gyrus | 32 | 6 | 42 | neg |
|  |  |  | Right Cingulate Gyrus | 14 | 28 | 30 | neg |
|  | p4R | 2, -36, 35 | Right Middle Frontal Gyrus | 32 | 0 | 42 | neg |
|  |  |  | Right Superior Frontal Gyrus | 28 | 46 | -20 | neg |
|  |  |  | Right Inferior Frontal Gyrus | 26 | 16 | -14 | neg |
|  |  |  | Right Middle Frontal Gyrus | 34 | 38 | 18 | neg |
|  |  |  | Right Anterior Cingulate | 8 | 22 | 28 | neg |
|  | p6R | 2, -47, 58 | Left Medial Frontal Gyrus | -4 | 60 | 24 | neg |
| C |  |  | Right Superior Frontal Gyrus | 26 | 44 | 34 | neg |
|  |  |  | Left Superior Frontal Gyrus | -14 | 34 | 42 | neg |
|  | p14L | -2, -64, 45 | Right Precentral Gyrus | 40 | -2 | 46 | neg |
|  |  |  | Left Middle Frontal Gyrus | -36 | -6 | 58 | neg |
|  |  |  | Left Inferior Frontal Gyrus | -56 | 8 | 26 | neg |
|  | p14R | 2, -64, 45 | Right Precentral Gyrus | 40 | -2 | 46 | neg |
|  |  |  | Left Middle Frontal Gyrus | -28 | -10 | 50 | neg |
|  |  |  | Left Medial Frontal Gyrus | 0 | -4 | 64 | neg |
|  | i9L | -5, 25, 10 | Left Substantia Nigra | -12 | -18 | -6 | pos |
|  |  |  | Left Cerebellar Tonsil | -8 | -46 | -42 | pos |
|  | s1L | -5, -10, 47 | Right Cingulate Gyrus | 16 | -50 | 28 | pos |
|  |  |  | Right Angular Gyrus | 48 | -70 | 36 | pos |
|  |  |  | Left Supramarginal Gyrus | -32 | -56 | 28 | pos |
|  | s1R | 5, -10, 47 | Right Supramarginal Gyrus | 70 | -46 | -14 | pos |
|  |  |  | Left Posterior Cingulate | -10 | -54 | 8 | pos |
|  |  |  | Right Cingulate Gyrus | 2 | -40 | 40 | pos |
|  |  |  | Left Uncus | -20 | -2 | -24 | pos |
|  |  |  | Right Inferior Temporal Gyrus | 62 | -12 | -18 | pos |
|  |  |  | Right Temporal Pole | 56 | 18 | -34 | pos |
|  |  |  | Left Fusiform Gyrus | -38 | -26 | -34 | pos |
|  | s3L | -5, 14, 42 | Right Postcentral Gyrus | 70 | -20 | 32 | pos |
|  |  |  | Right Supramarginal Gyrus | 44 | -42 | 30 | pos |
|  |  |  | Right Superior Temporal Gyrus | 58 | -18 | 4 | pos |
|  | s3R | 5, 14, 42 | Right Postcentral Gyrus | 66 | -20 | 36 | pos |
|  | s5L | -5, 24, 28 | Left Anterior Cingulate | -6 | 24 | 14 | pos |
|  | s7L | -5, 47, 11 | Left Dentate | -16 | -50 | -34 | pos |
|  | s7R | 5, 47, 11 | Right Substantia Nigra | 14 | -20 | -14 | pos |
|  |  |  | Right Uvula | 6 | -66 | -36 | pos |
|  |  |  | Left Insula | -40 | -10 | -12 | pos |
|  |  |  | Left Thalamus | -14 | -8 | 6 | pos |
|  |  |  | Left Cerebellar Tonsil | -26 | -50 | -42 | pos |
|  |  |  | Left Culmen | -12 | -34 | -20 | pos |
|  | p4L | -2, -36, 35 | Right Uvula | 30 | -82 | -36 | pos |
|  |  |  | Left Pyramis | -6 | -84 | -32 | pos |
|  | p4R | 2, -36, 35 | Left Paracentral Lobule | -20 | -42 | 54 | pos |
|  |  |  | Right Medial Frontal Gyrus | 16 | -8 | 52 | pos |
|  |  |  | Left Superior Frontal Gyrus | -16 | -16 | 70 | pos |
|  | p6L | -2, -47, 58 | Right Precuneus | 12 | -78 | 38 | pos |
|  |  |  | Left Cuneus | -10 | -98 | 4 | pos |
|  |  |  | Right Cuneus | 4 | -64 | 6 | pos |
|  |  |  | Left Precuneus | -18 | -84 | 34 | pos |
|  |  |  | Left Lingual Gyrus | -28 | -66 | -8 | pos |
|  |  |  | Right Middle Occipital Gyrus | 36 | -74 | 12 | pos |
|  |  |  | Right Declive | 32 | -62 | -20 | pos |
|  | p6R | 2, -47, 58 | Left Cuneus | -10 | -100 | 4 | pos |
|  |  |  | Left Declive | -16 | -80 | -18 | pos |
|  | p14L | -2, -64, 45 | Right Inferior Occipital Gyrus | 16 | -94 | -14 | pos |
|  | p14R | 2, -64, 45 | Right Middle Occipital Gyrus | 24 | -98 | 6 | pos |
|  |  |  | Left Cuneus | -20 | -100 | 22 | pos |
|  |  |  | Left Lingual Gyrus | -16 | -102 | -12 | pos |
|  |  |  | Right Fusiform Gyrus | 24 | -86 | -22 | pos |
|  | p17L | -1, -78, 43 | Left Cingulate Gyrus | -6 | -22 | 26 | pos |
|  |  |  | Right Precuneus | 12 | -82 | 46 | pos |
|  | p17R | 1, -78, 43 | Right Precuneus | 12 | -82 | 46 | pos |
|  |  |  | Left Cingulate Gyrus | -6 | -22 | 26 | pos |
| E | i9L | -5, 25, 10 | Right Medial Frontal Gyrus | 8 | 34 | 40 | neg |
|  |  |  | Right Anterior Cingulate | 16 | 36 | 8 | neg |
|  |  |  | Left Medial Frontal Gyrus | -2 | 60 | 12 | neg |
|  | i9R | 5, 25, -10 | Right Inferior Frontal Gyrus | 56 | 14 | 28 | neg |
|  |  |  | Left Declive of Vermis | -2 | -68 | -28 | neg |
|  |  |  | Right Inferior Frontal Gyrus | 30 | 28 | 18 | neg |
|  | s1L | -5, -10, 47 | Left Precuneus | -10 | -80 | 38 | neg |
|  |  |  | Right Cuneus | 14 | -76 | 12 | neg |
|  |  |  | Left Lingual Gyrus | -24 | -68 | -2 | neg |
|  | s1R | 5, -10, 47 | Right Middle Temporal Gyrus | 70 | -46 | -14 | neg |
|  |  |  | Left Cuneus | -10 | -84 | 34 | neg |
|  | s3R | 5, 14, 42 | Left Inferior Parietal Lobule | -64 | -40 | 34 | neg |
| E |  |  | Right Red Nucleus | 8 | -18 | -8 | neg |
|  |  |  | Left Thalamus | -16 | -16 | 16 | neg |
|  |  |  | Right Lentiform Nucleus | 24 | 0 | 12 | neg |
|  | s5L | -5, 24, 28 | Right Superior Temporal Gyrus | 54 | -6 | -8 | neg |
|  |  |  | Left Culmen | -12 | -42 | -10 | neg |
|  |  |  | Right Culmen | 22 | -42 | -16 | neg |
|  |  |  | Right Lentiform Nucleus | 22 | -2 | -8 | neg |
|  | s5R | 5, 34, 28 | Left Frontal Operculum | -32 | 32 | 10 | neg |
|  |  |  | Right Insula | 36 | 2 | 2 | neg |
|  |  |  | Left Lentiform Nucleus | -28 | -4 | 12 | neg |
|  | s7L | -5, 47, 11 | Left Declive | -14 | -66 | -30 | neg |
|  |  |  | Right Cerebellar Tonsil | 16 | -56 | -42 | neg |
|  |  |  | Right Uvula | 24 | -88 | -36 | neg |
|  | p6L | -2, -47, 58 | Right Middle Occipital Gyrus | 20 | -94 | 10 | neg |
|  |  |  | Left Cuneus | -4 | -88 | 38 | neg |
|  |  |  | Left Cuneus | 16 | -84 | 2 | neg |
|  |  |  | Right Precuneus | 20 | -66 | 32 | neg |
|  |  |  | Right Cuneus | 8 | -62 | 4 | neg |
|  | p6R | 2, -47, 58 | Left Declive | -10 | -72 | -20 | neg |
|  |  |  | Right Cuneus | 12 | -88 | 2 | neg |
|  |  |  | Left Precuneus | -14 | -72 | 30 | neg |
|  |  |  | Right Precuneus | 10 | -84 | 44 | neg |
|  |  |  | Left Middle Occipital Gyrus | -30 | -94 | 12 | neg |
|  | p14L | -2, -64, 45 | Right Cuneus | 14 | -98 | 0 | neg |
|  |  |  | Left Middle Occipital Gyrus | -26 | -94 | -2 | neg |
|  | p14R | 2, -64, 45 | Left Middle Occipital Gyrus | -24 | -94 | 2 | neg |
|  | i9L | -5, 25, 10 | Right Lingual Gyrus | 24 | -80 | -4 | pos |
|  |  |  | Left Lateral Occipital Cortex | -26 | -66 | 22 | pos |
|  |  |  | Left Cuneus | -2 | -96 | 6 | pos |
|  |  |  | Left Middle Occipital Gyrus | 46 | -78 | 2 | pos |
|  | s1L | -5, -10, 47 | Right Postcentral Gyrus | 52 | -22 | 52 | pos |
|  | s3L | -5, 14, 42 | Right Cuneus | 8 | -92 | 22 | pos |
|  |  |  | Right Lingual Gyrus | 10 | -74 | -12 | pos |
|  | s3R | 5, 14, 42 | Right Middle Temporal Gyrus | 56 | 8 | -28 | pos |
|  |  |  | Left Inferior Temporal Gyrus | -60 | -6 | -20 | pos |
|  |  |  | Left Cingulate Gyrus | -4 | -60 | 28 | pos |
|  | s5R | 5, 34, 28 | Left Lingual Gyrus | -24 | -80 | -6 | pos |
|  |  |  | Left Cuneus | -24 | -68 | 22 | pos |
|  | p4L | -2, -36, 35 | Right Culmen | 6 | -44 | -24 | pos |
|  | p4R | 2, -36, 35 | Right Cerebellar Lingual | 6 | -46 | -24 | pos |
|  | p6L | -2, -47, 58 | Right Superior Temporal Gyrus | 72 | -26 | 8 | pos |
|  |  |  | Right Postcentral Gyrus | 34 | -32 | 46 | pos |
|  |  |  | Right Postcentral Gyrus | 10 | -42 | 66 | pos |
|  | p6R | 2, -47, 58 | Right Postcentral Gyrus | 22 | 32 | 70 | pos |
|  |  |  | Right Superior Temporal Gyrus | 70 | -20 | 4 | pos |
|  |  |  | Left Precentral Gyrus | -36 | -16 | 50 | pos |
|  |  |  | Right Supramarginal Gyrus | 62 | -36 | 50 | pos |
|  |  |  | Right Precentral Gyrus | 34 | -18 | 46 | pos |
|  |  |  | Right Inferior Frontal Gyrus | 56 | 12 | 30 | pos |
|  |  |  | Left Inferior Frontal Gyrus | -58 | 6 | 28 | pos |
|  |  |  | Right Insula | 34 | -32 | 16 | pos |
|  | p14L | -2, -64, 45 | Right Precentral Gyrus | 40 | -2 | 46 | pos |
|  |  |  | Right Lingual Gyrus | 34 | -56 | 4 | pos |
|  |  |  | Right Culmen | 18 | -36 | -14 | pos |
|  | p14R | 2, -64, 45 | Right Precentral Gyrus | 40 | -4 | 36 | pos |
|  |  |  | Left Culmen | -14 | -48 | -14 | pos |
|  |  |  | Left Cuneus | -16 | -74 | 14 | pos |
|  | p17L | -1, -78, 43 | Right Precentral Gyrus | 38 | 0 | 40 | pos |
|  |  |  | Left Middle Occipital Gyrus | -40 | -68 | -6 | pos |
|  |  |  | Left Precentral Gyrus | -52 | -20 | 36 | pos |
|  |  |  | Right Precentral Gyrus | 62 | -2 | 16 | pos |
|  |  |  | Right Cuneus | 4 | -84 | 16 | pos |
|  |  |  | Left Declive of Vermis | -2 | -68 | -30 | pos |
|  |  |  | Right Middle Occipital Gyrus | 34 | -78 | 6 | pos |
|  |  |  | Left Precentral Gyrus | -52 | -8 | 8 | pos |
|  | p17R | 1, -78, 43 | Right Precentral Gyrus | 38 | 0 | 40 | pos |
|  |  |  | Left Declive | -14 | -60 | -16 | pos |
|  |  |  | Left Postcentral Gyrus | -40 | -22 | 30 | pos |
| E |  |  | Right Precentral Gyrus | 62 | -2 | 16 | pos |
|  |  |  | Right Fusiform Gyrus | 34 | -68 | -16 | pos |
|  |  |  | Right Cuneus | 4 | -86 | 16 | pos |
|  |  |  | Left Middle Occipital Gyrus | -36 | -82 | -6 | pos |
| N | s1R | 5, -10, 47 | Right Declive | 20 | -80 | -30 | neg |
|  | s3L | -5, 14, 42 | Left Medial Frontal Gyrus | -6 | 24 | -18 | neg |
|  |  |  | Left Anterior Cingulate | -6 | 50 | 0 | neg |
|  | p4L | -2, -36, 35 | Right Insula | 42 | 2 | 14 | neg |
|  |  |  | Right Insula | 26 | 16 | -10 | neg |
|  | p4R | 2, -36, 35 | Right Superior Frontal Gyrus | 24 | 58 | -4 | neg |
|  |  |  | Right Middle Frontal Gyrus | 48 | 50 | 18 | neg |
|  | p14L | -2, -64, 45 | Right Middle Frontal Gyrus | 38 | 44 | 18 | neg |
|  |  |  | Right Insula | 44 | 0 | -6 | neg |
|  | p14R | 2, -64, 45 | Right Inferior Frontal Gyrus | 44 | 16 | 16 | neg |
|  |  |  | Right Middle Frontal Gyrus | 38 | 46 | 20 | neg |
|  |  |  | Right Insula | 40 | -2 | -20 | neg |
|  | s1R | 5, -10, 47 | Right Inferior Frontal Gyrus | 48 | 30 | 10 | pos |
|  |  |  | Right Precuneus | 22 | -60 | 20 | pos |
|  |  |  | Right Inferior Frontal Gyrus | 50 | 8 | 32 | pos |
|  |  |  | Left Cingulate Gyrus | -10 | -56 | 28 | pos |
|  | s3L | -5, 14, 42 | Right Lingual Gyrus | 18 | -82 | -10 | pos |
|  | s7L | -5, 47, 11 | Right Declive | 32 | -64 | -18 | pos |
|  | s7R | 5, 47, 11 | Left Declive of Vermis | 0 | -70 | -28 | pos |
|  | p4L | -2, -36, 35 | Left Medial Frontal Gyrus | -2 | 44 | 42 | pos |
|  |  |  | Right Posterior Cingulate | 4 | -50 | 12 | pos |
|  |  |  | Right Frontal Pole | 2 | 66 | 0 | pos |
|  |  |  | Left Precuneus | -20 | -44 | 34 | pos |
|  | p4R | 2, -36, 35 | Left Medial Frontal Gyrus | 0 | 62 | 26 | pos |
|  |  |  | Left Frontal Pole | 2 | 66 | -8 | pos |
|  | p6L | -2, -47, 58 | Left Lingual Gyrus | -14 | -78 | -8 | pos |
|  |  |  | Right Precuneus | 20 | -84 | 36 | pos |
|  |  |  | Right Lingual Gyrus | 18 | -74 | -10 | pos |
|  |  |  | Right Postcentral Gyrus | 40 | -38 | 58 | pos |
|  | p6R | 2, -47, 58 | Right Lingual Gyrus | 18 | -74 | -10 | pos |
|  |  |  | Right Cuneus | 20 | -88 | 36 | pos |
|  |  |  | Right Parahippocampal Gyrus | 26 | -44 | -14 | pos |
|  | p14L | -2, -64, 45 | Right Middle Temporal Gyrus | 74 | -32 | 0 | pos |
|  |  |  | Left Postcentral Gyrus | -42 | -22 | 46 | pos |
|  |  |  | Right Middle Temporal Gyrus | 68 | -4 | -10 | pos |
|  | p14R | 2, -64, 45 | Left Middle Temporal Gyrus | -72 | -46 | -8 | pos |
|  |  |  | Left Middle Temporal Gyrus | -46 | -24 | -14 | pos |
|  | p17L | -1, -78, 43 | Left Postcentral Gyrus | -42 | -22 | 42 | pos |
| O | i9L | -5, 25, 10 | Right Precuneus | 20 | -70 | 40 | neg |
|  |  |  | Right Middle Temporal Gyrus | 44 | -78 | 20 | neg |
|  | i9R | 5, 25, -10 | Left Inferior Frontal Gyrus | -30 | 26 | -2 | neg |
|  |  |  | Left Middle Frontal Gyrus | -16 | 50 | -20 | neg |
|  |  |  | Left Rectal Gyrus | -8 | 18 | -26 | neg |
|  | s1L | -5, -10, 47 | Right Medial Frontal Gyrus | 16 | -10 | 60 | neg |
|  |  |  | Left Superior Frontal Gyrus | -18 | -6 | 58 | neg |
|  | s3L | -5, 14, 42 | Left Precentral Gyrus | -14 | -24 | 68 | neg |
|  |  |  | Right Postcentral Gyrus | 24 | -36 | 52 | neg |
|  |  |  | Left Precuneus | -4 | -48 | 50 | neg |
|  |  |  | Left Precentral Gyrus | -44 | -14 | 60 | neg |
|  | s7L | -5, 47, 11 | Right Frontal Pole | 42 | 64 | -2 | neg |
|  |  |  | Left Inferior Frontal Gyrus | -52 | 32 | 16 | neg |
|  |  |  | Right Inferior Frontal Gyrus | 42 | 28 | 14 | neg |
|  | s7R | 5, 47, 11 | Left Inferior Frontal Gyrus | -34 | 22 | 20 | neg |
|  | p17L | -1, -78, 43 | Left Postcentral Gyrus | -50 | -26 | 40 | neg |
|  |  |  | Right Medial Frontal Gyrus | 14 | -26 | 58 | neg |
|  |  |  | Right Precentral Gyrus | 44 | -12 | 58 | neg |
|  | p17R | 1, -78, 43 | Left Postcentral Gyrus | -50 | -28 | 40 | neg |
|  |  |  | Right Postcentral Gyrus | 52 | -16 | 26 | neg |
|  |  |  | Left Transverse Temporal Gyrus | -60 | -12 | 14 | neg |
|  |  |  | Right Precentral Gyrus | 28 | -20 | 72 | neg |
|  | i9L | -5, 25, 10 | Right Medial Frontal Gyrus | 2 | 48 | 26 | pos |
|  |  |  | Left Precentral Gyrus | -36 | -18 | 40 | pos |
|  |  |  | Left Precentral Gyrus | -54 | -6 | 14 | pos |
| O |  |  | Right Medial Frontal Gyrus | 10 | 54 | -6 | pos |
|  | i9R | 5, 25, -10 | Right Medial Frontal Gyrus | 2 | 48 | 26 | pos |
|  |  |  | Right Medial Frontal Gyrus | 6 | 54 | -4 | pos |
|  | s1L | -5, -10, 47 | Left Superior Frontal Gyrus | -14 | 58 | 26 | pos |
|  |  |  | Right Medial Frontal Gyrus | 2 | 54 | -2 | pos |
|  |  |  | Left Occipital Pole | -26 | -100 | 22 | pos |
|  |  |  | Left Middle Temporal Gyrus | -58 | -14 | -14 | pos |
|  |  |  | Left Rectal Gyrus | -2 | 32 | -30 | pos |
|  |  |  | Left Inferior Occipital Gyrus | -40 | -92 | -6 | pos |
|  |  |  | Right Anterior Cingulate | 14 | 36 | 20 | pos |
|  |  |  | Left Superior Temporal Gyrus | -54 | -44 | 10 | pos |
|  |  |  | Left Cuneus | 0 | -92 | 6 | pos |
|  | s1R | 5, -10, 47 | Right Inferior Temporal Gyrus | 60 | -12 | -18 | pos |
|  |  |  | Right Medial Frontal Gyrus | 2 | 50 | -6 | pos |
|  |  |  | Right Middle Temporal Gyrus | 62 | -36 | 2 | pos |
|  |  |  | Left Superior Temporal Gyrus | -44 | -50 | 14 | pos |
|  |  |  | Left Middle Temporal Gyrus | -54 | -20 | -10 | pos |
|  |  |  | Left Middle Frontal Gyrus | -54 | 32 | 18 | pos |
|  |  |  | Left Inferior Frontal Gyrus | -28 | 18 | -12 | pos |
|  |  |  | Right Temporal Pole | 54 | 18 | -36 | pos |
|  | s5L | -5, 24, 28 | Left Cingulate Gyrus | -4 | -33 | 36 | pos |
|  |  |  | Right Cingulate Gyrus | 16 | 2 | 32 | pos |
|  | s7L | -5, 47, 11 | Left Superior Frontal Gyrus | -18 | 66 | 2 | pos |
|  |  |  | Right Medial Frontal Gyrus | 10 | 56 | -8 | pos |
|  |  |  | Right Cingulate Gyrus | 16 | -52 | 26 | pos |
|  |  |  | Left Subcallosal Gyrus | 0 | 22 | -14 | pos |
|  | p6R | 2, -47, 58 | Left Lingual Gyrus | -10 | -92 | -8 | pos |
|  | p14R | 2, -64, 45 | Right Pyramis | 22 | -78 | -40 | pos |
|  | p17L | -1, -78, 43 | Right Precuneus | 12 | -82 | 46 | pos |
|  |  |  | Right Precuneus | 24 | -62 | 24 | pos |
|  | p17R | 1, -78, 43 | Right Precuneus | 12 | -82 | 46 | pos |
|  |  |  | Right Precuneus | 24 | -62 | 24 | pos |

Table of local peaks associated with each seed-RSFC relationship for each domain of personality. Coordinates are in standard MNI152 space. Seed locations are shown in Figure 1. Behavior refers to behavioral relationships: positive relationships are stronger with higher personality domain scores, negative relationships are stronger with lower personality domain scores. pos = positive relationship; neg = negative relationship. N = Neuroticism; E = Extraversion; O = Openness; A = Agreeableness; C = Conscientiousness.
